# Pulmonary tuberculosis case notification and burden of drug resistance among children under 15 years of age in Ethiopia: sub-analysis from third-round drug resistance tuberculosis survey

**DOI:** 10.1186/s12887-023-04240-6

**Published:** 2023-08-24

**Authors:** Yeshiwork Abebaw, Markos Abebe, Habteyes Hailu Tola, Zemedu Mehammed, Muluwork Getahun, Dinka Fikadu Gamtesa, Getu Diriba, Michael Hailu, Hailegiorgis Yirgu, Anandi Nayan Sheth, Rahel Argaw, Woldaregay Erku Abegaz

**Affiliations:** 1 Department of Microbiology, Immunology and Parasitology, College of Health Sciences, Addis Ababa University and Ethiopian Public Health Institute, Addis Ababa, Ethiopia; 2 Ethiopian Public Health Institute, Addis Ababa, Ethiopia; 3 Armauer Hansen Research Institute, Addis Ababa, Ethiopia; 4 KNCV Tuberculosis Foundation, Addis Ababa, Ethiopia; 5 Division of Infectious Diseases, Department of Medicine, Emory University School of Medicine, Atlanta, Georgia; 6 Department of Pediatrics and Child Health, College of Health Sciences, Addis Ababa University, Addis Ababa, Ethiopia

**Keywords:** Children, Tuberculosis, Case notification, Drug resistance

## Abstract

**Introduction:**

Data on the burden of bacteriologically confirmed childhood Tuberculosis (PTB) and drug-resistant TB in Ethiopia is limited due to difficulties related to its diagnosis in this population. Therefore, this study aimed to assess bacteriologically confirmed childhood PTB Case Notification Rates (CNRs) and the burden of Drug Resistant-Tuberculosis among children in Ethiopia.

**Method:**

Retrospective secondary clinical and laboratory data were obtained from 3rd round national DR-TB survey which was conducted between August 2017 and January 2019. We used IBM SPSS 24 for sub-analysis of 3rd round Drug Resistant-Tuberculosis data. Descriptive statistics were used in computing the association between the sociodemographic characteristics and PTB CNRs, and the strength of the associations was determined using binary logistic regression with Odds ratios (OR) with a 95% confidence interval (CI).

**Result:**

Overall, 102 bacteriologically confirmed childhood PTB cases were identified with a median age of 12 (range 1–14) years. Of these, 54 (52.9%) were females and 81 (79.4%) lived in rural areas. HIV-TB co-infection cases were 5/102 (4.3%) and the majority (98%) of cases were newly diagnosed children. Nationally, the incidence of bacteriologically confirmed childhood PTB was calculated to be 5.1 per 100,000 children. The burden of Drug Resistant-Tuberculosis to at least one of the five first-line anti-TB drugs tested was five (6.5%) cases and one (1.3%) was found to be a Multi-drug resistant tuberculosis case. Drug-resistant tuberculosis was significantly associated with the age group 10–14 years (P = 0.002; [AOR] 29.76; [95% CI, 3.51-252.64]) and children living in urban areas (P = 0.027; [AOR] 5.76; 95% CI, 1.22–27.09).

**Conclusion:**

Bacteriologically confirmed childhood PTB cases increased as the age of the children increased. Most of the bacteriologically confirmed childhood PTB and the identified drug Resistant-Tuberculosis cases were new cases. Also, rural children were more affected by TB than their urban, counterparts Drug Resistant-Tuberculosis was higher in urban resident children.

**Supplementary Information:**

The online version contains supplementary material available at 10.1186/s12887-023-04240-6.

## Introduction

Recently, tuberculosis has been recognized as a silent epidemic disease in children [1]. Of the 10 million estimated new TB cases that occurred in 2021, 1.1 million were children [2]. Moreover, it has been reported that an estimated 33,000 children developed Multi-drug resistant tuberculosis (MDR-TB) each year across the world [3]. A 2015 modeling study reported that a high burden of childhood DR-TB was observed in the European and Western Pacific World Health Organization (WHO) regions due to the presence of high proportions of Drug Resistant-Tuberculosis (DR-TB) among the general population in those regions [4].

Based on the previous survey, Ethiopia was under high MDR-TB burden country (2.7% among new and 14% among previously treated TB cases) [5]. However, with the current 3rd round DR-TB surveys, the country was excluded from the 30 high MDR-TB burden counties[6].

In Ethiopia, the burden of childhood TB was estimated 24,000 in 2017 [7]. However, in the first population-based national tuberculosis prevalence survey of Ethiopia, children were not included due to the technical difficulty of screening and diagnosing TB cases in this population [5, 8]. Interestingly, after the introduction of GeneXpert MTB/RIF assay (Cepheid, Sunnyvale, CA, USA) in the country, studies have been conducted that show the improvement of TB case and rifampicin resistance tuberculosis cases detection from children with TB [9–11]. Even then, diagnosis of childhood TB is still challenging because of having non-specific symptoms and difficulty in detecting paucibacillary TB in children. As a result, the country recently endorsed Xpert MTB/RIF Ultra since it has a better ability to detect paucibacillary TB compared to GeneXpert MTB/RIF assay [12].

Ethiopia adopted a national childhood TB roadmap in 2015 following the global childhood TB roadmap to improve case detection and management [13]. So far, Childhood TB treatment success in Ethiopia ranged from 78.0 to 92.6% [14, 15]. The country’s performance of BCG vaccination coverage increased from year to year (by 47.6% % during the years 2002 to 2019) [16] although, the efficacy of BCG was low[17].

In this connection, it is of paramount importance for TB programs to understand the epidemiology of childhood TB as it may give an opportunity for better prevention and control of the disease in this population and consequently in the community at large because childhood TB is a marker of recent transmission [18]. However, there is a scarcity of studies on the burden of childhood TB, and more so on bacteriologically confirmed childhood PTB and DR-TB, case notification reports at the national level. Therefore, this study was conducted to determine the level of childhood TB case notification and the burden of DR-TB among Ethiopian children registered for bacteriologically confirmed Tuberculosis cases during the third-round drug resistance tuberculosis survey.

## Methods

### Study design and study area

Retrospective secondary clinical and laboratory data were obtained from the 3rd round national DR-TB survey which was conducted between August 2017 and January 2019 [19]. The survey was conducted using samples collected from 199 health facilities that are considered to be national representatives from all over the country. However, bacteriologically confirmed childhood PTB cases were reported only from 62/199 (31%) health facilities. The survey enrolled a total of 2560 study participants of whom 119 of these were children under 15 years of age, who were the subjects for this study.

### Eligibility criteria

**Fig. 1:**
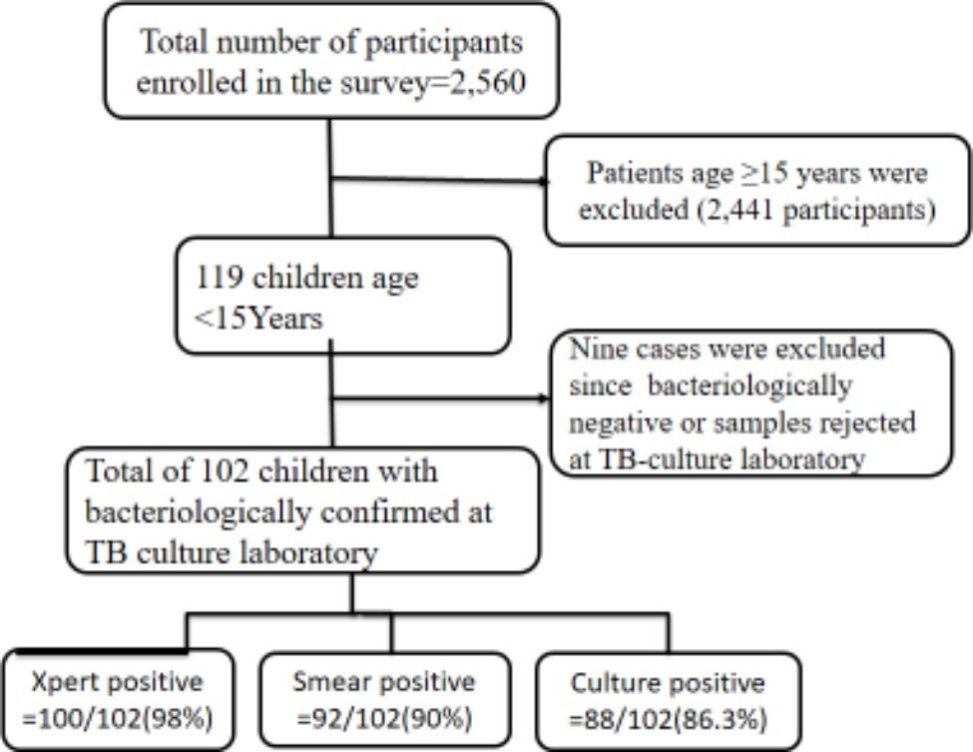
Workflow of participants’ enrolment in this study

While the 3rd round national DR-TB survey included both adults and children diagnosed with pulmonary tuberculosis, this study was focused only on children aged < 15. A patient was enrolled in the study if he/she was a bacteriologically confirmed pulmonary tuberculosis case at selected the health facility but excluded from the survey if he/she was treated for TB for more than one week before the study period. Additionally, enrollment of a participant was terminated if his/her sputum sample was of poor quality or if it was found bacteriologically negative at the TB-culture laboratory. Therefore, from 119 Children enrolled in the survey, only 102 were identified as bacteriologically confirmed childhood PTB at the TB-culture laboratory (Fig. 1). Thus, the entire 102 childhood bacteriologically confirmed childhood PTB cases were eligible for this study.

### Laboratory tests

At the selected diagnostic health facilities, all presumptive TB cases were screened for pulmonary TB positivity based on the national TB diagnostic algorithm, and enrolled into the study if the patient is positive for tuberculosis either by GeneXpert MTB/RIF and/or smear microscopy using the fluorescent staining method. Then about 2-5ml of morning sputum sample was collected with a 50ml of serial falcon tube from each participant and sent to the nearest TB-culture laboratory for further investigation. The samples were transported in a cold chain by using a standard triple packaging system for two days through the postal system. Bacteriologically confirmed childhood PTB was determined at least with one or more of the following TB diagnostic tests at TB-culture laboratories; smear microscopy using the fluorescent staining method, GeneXpert MTB/RIF from sediment, and TB-culture with Mycobacteria Growth Indicator Tube (MGIT) (Bactec, Inc 960 system) and Lowenstein-Jensen (LJ) medium.

### Fluorescent staining method

For the presence of the Acid-Fast Bacilli (AFB), spot samples at a selected health facility and sediment of morning sputum from the same patient at the TB-culture laboratory were tested by fluorescent staining method [20]. The prepared slides were stained with 0.1% auramine for 20 min and then rinsed with water. After rising 0.5% of acid-alcohol was added and left for 3 min to allow decolorization. Then, it was rinsed again and stained with 1% of Potassium permanganate solution for 1 min. After one minute of staining the slide was dried after which it was examined using LED microscopy at 40 X objectives. The bacilli appear as slender bright yellow luminous rods in a dark field, and the results were graded as per the guidelines of the International Union Against Tuberculosis and Lung Disease (IUATLD) scale[20].

### Xpert MTB/RIF assay

The GeneXpert MTB/RIF assay was performed as described previously [20] [21] from direct sputum and sediment at the selected health facilities and TB-culture laboratory, respectively. Briefly, direct sputum was processed from two parts of the sample reagent to one part of the sputum sample, making the ratio 2:1. While the sediment was performed using a 3:1 ratio of sample reagent added to the processed sputum sample (sediment). Then the specimen container was closed and manually agitated twice during a 15-min incubation period at room temperature. Then, 2 ml of the homogenized sample was transferred to the test cartridge. Finally, the specimen containing the cartridge was loaded onto the Xpert machine, and the results were displayed after 2 h on the Xpert computer.

### Sputum culture

Sputum was processed for culture by digesting and decontaminating the sputum with the N-acetyl-L-cysteine-sodium hydroxide method and then inoculated onto both LJ and MGIT [22]. The LJ medium culture was incubated at 37 ^o^C and inspected weekly for eight weeks to see the growth of Mycobacterium colonies. If there no growth was observed at the end of the eighth week, the result was considered negative [22–24].

The MGIT tubes were incubated at 37 °C in the BACTEC MGIT 960 instrument and monitored automatically every 60 min for a maximum of 42 days. At 42 days of incubation, the result was flagged as negative on the machine. All the positive tubes were further confirmed by using blood agar and Zehil Nelson (ZN) staining to assess if contaminants exist. Blood agar-negative, smear-positive samples were further confirmed by rapid immuno-chromatographic assay (SD bioline) for the presence of mycobacterium tuberculosis complex [23, 24].

### A phenotypic drug susceptibility test

A phenotypic drug susceptibility test (DST) against first-line drugs (Streptomycin, Isoniazid, Rifampicin, Ethambutol (SIRE); and Pyrazinamide (PZA) was also conducted based on WHO recommended critical concentrations method using MGIT [25]. DST was conducted according to previously published protocol using MGIT (Bactec, Inc) 960 system [22]. A known concentration of the drug was added to an MGIT, along with the specimen, and growth was compared with a drug-free control of the same Specimen.

### Definition

WHO’s age stratification for epidemiological studies was used for the purpose of this study, where children were defined as persons whose age is less than 15 years [26]. A bacteriologically confirmed childhood PTB refers to the presence of TB bacilli from sputum by using one or more TB diagnostic tests (smear microscopy, culture, and/ or GeneXpert MTB/RIF). First-line anti-TB drugs included isoniazid, rifampin, ethambutol, pyrazinamide, and streptomycin. Drug-resistant tuberculosis refers to any resistance to first-line anti-TB drugs. Mono-resistance refers to resistance to any one of the first-line anti-TB drugs only. Multidrug resistance refers to resistance to at least both isoniazid and rifampicin.

### Data extraction and cleaning

The 3rd round of national DR-TB survey data collected from August 2017 to January 2019 was used as the data source. We received this data file in an Excel spreadsheet from the Ethiopian Public Health Institute National tuberculosis reference laboratory. The received Data file was cross-checked for accuracy with the collected questionnaire and laboratory registration logbook of the survey before analysis. For the purpose of this study, the study participants’ data were disaggregated by age (0–4, 5–9, and 10–14 years), history of treatment (new or previously treated), sex (male or female), and drug resistance profile.

### Data analysis

We used IBM SPSS 24 for sub-analysis of 3rd round DR-TB data. Descriptive statistics were used to present the socio-demographic and clinical characteristics of the study participants. We obtained the 2017 population estimation data on children under 15 of age for each study area from the Central Statistical Agency of Ethiopia (CSA) [27] and computed the bacteriologically confirmed childhood PTB CNRs using the data therefrom. Accordingly, the estimation of the number of under 15 children who received healthcare services from a particular region was calculated using the following formula: (X/Y)*Z, where X = a number of selected healthcare service provider institutions in the region; Y = the total number of healthcare provider institutions in the region; and Z = total number of under 15 children in the region according to the 2017 CSA survey. We also compared the sociodemographic and clinical characteristics of patients with pan-susceptible versus drug-resistant tuberculosis and calculated the Odds ratios (OR) with a 95% confidence interval (CI) by using binary logistic regression. P values less than 0.05 were considered significant.

## Results

### Socio-demographic characteristics of the study participants

The study identified a total of 102 bacteriologically confirmed childhood PTB during the 3rd round DR-TB survey, which was held country-wide between August 2017 and January 2019. Of the 102 bacteriologically confirmed childhood PTB cases, 48 (47.1%) cases were males, and 81 (79.4%) were rural residents. HIV-TB co-infection cases were 5/102 (4.3%). The median age of the participating children was 12 (range 1–14) years old; most of the cases (77%) were in the age group 10–14 years old. Moreover, 52 (51%) of the cases had previous TB contact (Table 1).

**Table 1:** Clinical and sociodemographic characteristics of children enrolled in the national DR-TB survey from August 2017 to January 2019 (n = 102)

Characteristic		Children Age < 15 n (%)
Age	Median age(range)	12(1–14)
	0–4	6(6)
	5–9	17(17)
	10–14	79(77)
Sex	Male	48(47.1)
	Female	54(52.9)
Residence	Rural	81(79.4)
	Urban	21(20.6)
HIV status	Negative	95(93.1)
	Positive	5(4.9)
	Unknown	2(2)
Previous history of TB treatment	New	99(97.1)
	Previously treated	3(2.9)
known contact	Yes	52(51)
	No	50(49)

### The burden of bacteriologically confirmed childhood pulmonary tuberculosis

Only 62(32%) of the 199 selected health facilities were able to report bacteriologically confirmed childhood PTB. Although health facilities from Afar (two), Dire-Dawa (three), and Benishangul-Gumuz (four) regional states were included in the survey, no case was reported from them, despite the fact that there were an estimated 62,1283 children in Afar, 13,7451 in Dire Dewa and 41,9729 in Benishangul-Gumuz who could have been served by the respective selected health facilities.

Estimation of the national cumulative incidence rate of bacteriologically confirmed childhood PTB during the period between August 2017 and January 2019 was computed to be 5.1 per 100,000 children (Table 2). The cumulative incidence rate of bacteriologically confirmed childhood PTB notification differed by region. The highest TB CNRs were observed in Gambela regional state (11.57 per 100,000 children) followed by SNNP regional state (8.1 per 100, 0000 children). The lowest incidence was documented in Tigray regional state (2.45 per 100, 0000 children) (Table 2).

**Table 2:** National and regional cumulative incidence of bacteriologically confirmed childhood pulmonary tuberculosis in Ethiopia by age group and sex from August 2017 to January 2019 (N = 99)

Region	HF report	Regional and National Total under 15 children			Age group									Under 15 children by sex					
					0–4			5–9			10–14			Male			Female		
		Population	Total cumulative new Cases	cases per 10^5^	population	cases	cases per 10^5^	population	Cases	cases per 10^5^	population	Cases	cases per 10^5^	population	cases	cases per 10^5^	population	cases	cases per 10^5^
National	62/199	1,945,009	99	5.1	704,762	5	0.71	648,490	15	2.31	591,758	79	13.35	985,949	48	4.87	959,062	51	5.32
Tigray	2/10	81,506	2	2.45	28,771	0	0	26,972	0	0	25,764	2	7.76	41,415	2	4.83	40,089	0	0
Amhara	6/39	321,109	8	2.49	89,952	2	2.22	87,898	1	1.14	85,625	5	5.84	133,281	4	3	130,210	4	3.1
Oromia	31/80	841,750	44	5.23	302,806	2	0.66	275,682	6	2.2	263,262	36	13.67	426,286	19	4.5	415,464	25	6.02
Somali	1/2	25,417	2	7.87	10,392	0	0	9274	0	0	5751	2	34.8	13,071	0	0	12,346	1	8.1
SNNPR	18/44	456,314	37	8.1	162,904	1	0.61	146,554	8	5.46	146,857	28	19.16	230,206	21	9.12	226,108	16	7.16
Gambella	1/2	8638	1	11.57	3071	0	0	2849	0	0	2719	1	37	4438	0	0	4201	1	24
Harari	1/2	15,954	1	6.3	5512	0	0	5295	0	0	5147	1	19.43	8,150	0	0	7,803	1	12.82
Addis Ababa	2/11	90,111	4	4.44	37,640	0	0	31,878	0	0	20,594	4	19.42	44,952	2	4.45	45,153	2	4.43

Abbreviations: HF = Health Facility; SNNPR = Southern Nations Nationalities & Peoples Region

Age-dependent analyses showed that the national incidence of bacteriologically confirmed childhood PTB notification rate was lowest (0.71 per 100,0000 children) among children under five years of age and increased as the age of the children increased, the maximum being among the 10–14 years age group (13.35 per 100,0000 children). This nationally observed age-dependent direct association of childhood TB was also observed in the overwhelming majority of regional states, except in the Amhara regional state, where more TB CNRs were observed among children younger than five years of age compared to those in the age range of 5 to 9 years (2.22 vs. 1.14 per 100,000).

The national incidence of TB CNRs by gender was slightly higher among female than male children (5.32 vs. 4.87 per 100,000). The same pattern was observed among the regional states also, except for Tigray (4.83 vs. 0.00 per 100,000), Southern Nations Nationalities & Peoples Region (SNNPR) (9.12 vs. 7.16 per 100,000), and Addis Ababa (4.45 Vs 4.43 per 100,00 children) where it was slightly higher among males than females.

### The burden of drug-resistant childhood pulmonary TB

From the 102 identified childhood TB cases, it was possible to resuscitate only 77 TB isolates for first-line phenotypic drug susceptibility testing (DST) (75 of them were from new and two were from previously treated cases). The burden of DR-TB to any one of the five first-line anti-TB drugs tested (Isoniazid, Rifampin, Ethambutol, Streptomycin, and Pyrazinamide was 5/77 (6.5%). An interesting observation in this study was that all the identified drug-resistant isolates were only from children who had no previous treatment history (i.e., among 5/75; 6.6% of newly infected). Moreover, the burden of MDR-TB was 1.3% while mono-resistant isolates of isoniazid and streptomycin were 2.6% and 1.3%, respectively (Table 3).

**Table 3:** Drug susceptibility results for childhood TB cases among children aged 0–14 years (N = 77)

Drug resistance profile of Isolates	*New cases = 77 n (%)
Drug resistance to at least one first-line anti-TB Drug	5/77(6.5)
INH	2/77(2.6)
RIF	0
EMB	0
PZA	0
STR	1/77(1.3)
STR + INH	1/77(1.3)
RIF + INH + STR + EMB + PZA	1/77(1.3)

Abbreviations: n = Number of participants; INH = Isoniazid; RIF = Rifampin; EMB = Ethambutol; STR = Streptomycin; PZA = Pyrazinamide

In this study, DR-TB was significantly associated with the age group 10–14 years ((P value < 0.05 [AOR] 29.76; [95% CI, 3.51-252.64]). Also, DR-TB was higher among females (3/5 (60%)), although not statistically significant (P value = 0.76). A significant association was observed between DR-TB and place of residence, where children living in urban areas were more prone to DR-TB than those living in rural ([AOR] 5.76; 95% CI, 1.22–27.09 (p-value = 0.027) (Table 4).

**Table 4:** Comparison of Pan-susceptible TB with DR-TB by socio-demographic characteristics of participant children using logistic regression models. (Pan = susceptible = 72); DR-TB = 5)

Socio-demographic		DST result		Crude OR (95% CI)	p-value	Adjusted OR (95% CI)	p-value
		Pan-susceptible TB (n = 72) (100%)	DR-TB(n = 5) (100%)				
Age	**0–9**	15(20.8)	1(20)	1			
	**10–14**	57(79.2)	4(80)	14.25(5.17–39.27)	0.00	29.76(3.51-252.64	**0.002**
Sex	**Male**	37(51.4)	2(40)	1			
	**Female**	35(48.6)	3(60)	11.67(3.5–37.9)	0.00	0.75(0.13–4.54	0.76
Residence	**Urban**	13(18.1)	3(60)	29.5(7.21-120.73)	0.00	5.76(1.22–27.09)	**0.027**
	**Rural**	59(81.9)	2(40)	1			
Had TB Contact	**Yes**	34(47.2)	4(80)	8.5(3.0-23.95)	0.00	0.320 (0.05–2.05)	0.229
	No	38(52.8)	1(20)	1			

Abbreviations: n = Number of participants; DR = drug resistant; OR = Odds Ratio; CI = Confidence Interval

## Discussion

There is limited literature on the epidemiology and drug resistance pattern of childhood tuberculosis in Ethiopia [8]. This is the first report on a countrywide bacteriologically confirmed childhood PTB CNRs in the country.

A total of 102 nationally identified bacteriologically confirmed childhood PTB cases were included in this study. Of these 102 bacteriologically confirmed childhood PTB cases, only fewer (6/102; 5.9%) were among children younger than five years of age, which was higher than bacteriologically confirmed childhood PTB cases reported from southern Ethiopia (1.9%) [28]. But much lower than both clinically identified and bacteriologically confirmed cases reported from scattered locally focused previous studies: 21.8% from Addis Ababa [15], 30.4% from Tigray region [29], and 45.5% from southern Ethiopia [28]. Similar to the previous studies from the aforementioned Ethiopian regions, but in contrast to our finding, studies from other parts of Africa such as Zambia (61%) also showed a higher burden of TB among children younger than five years of age compared to children aged 5–14 years [30]. This disagreement between our findings and those from the other studies above may be because of the fact that our study included only children with bacteriologically confirmed childhood PTB cases while the other studies considered both bacteriologically confirmed and clinically diagnosed TB cases. This difference also highlights the challenges associated with the reliability of childhood TB diagnosis and decision-making merely based on clinical findings without considering bacteriological confirmation [31, 32].

Whereas bacteriologically-confirmed childhood pulmonary TB cases were slightly higher among female (52.9%) than male (47.1%) children in this study, with an incidence rate of 5.3 per 100,000 and 4.87 per 100,000 children, respectively, adult TB cases from the same 3rd round DR-TB survey and those reported from most previous studies, contrarily, demonstrated a male TB case predominance [33]. Similar to our finding, however, previous findings from Addis Ababa (55.4% vs. 44.6%) [15] and Zimbabwe (53% vs. 47%) [30] also reported a TB detection rate higher among females than males. The reason why more female than male children are affected could probably be due to female children staying at home taking care of their sick household adults more than their male counterparts. Nevertheless, this needs further investigation to definitely understand the true reasons behind such a lopsided TB detection rate among female children.

In this study, most of the children with confirmed TB were from rural areas (79.4%) which was similar to the finding from a study done in Debre Markos Referral Hospital, Northwest, Ethiopia[10]. In contrast, many other studies demonstrated that a high TB burden occurs in urban environments due to overcrowding, high HIV prevalence, and occupational transmission [33, 34]. However, the reports from these other studies, including even the one from the national TB program, took patients’ reporting sites into consideration during their data analyses instead of from patients’ places of residence [35]. Data on bacteriologically confirmed childhood PTB CNRs by place of residence (as is the case in this study) rather than a place of diagnosis (as the cases in the other studies mentioned above) may correctly identify the source of TB transmission more accurately [36].

Ethiopia is a high-burden country for HIV-TB co-infection [37]. We found that 4% of childhood tuberculosis cases in our study occurred among children with HIV co-infection, a value higher than that reported from a previous study in southern Ethiopia (2.3%) [28] but much lower than reported from Addis Ababa (28.2%) and other African countries (E.g., Togo (14.9%) [38]. The low rate of HIV-TB co-infection in our study may be related to the high number of cases occurring in rural areas (79.4%), where HIV prevalence is lower than that in urban areas of Ethiopia [39].

In the present study, almost all childhood TB cases (98%) were from newly diagnosed patients, which is similar to a previous report from south Ethiopia (98.2%) but slightly higher than the report from Addis Ababa (92%) [15]. This high childhood TB prevalence from newly diagnosed patients most likely indicates the presence of an ongoing transmission of tuberculosis within the community [40] since children are at higher risk of developing the disease within a short period of infection [41]. In this connection, it has already been established that the majority of children, especially those < 5 years of age and those with HIV co-infection, develop tuberculosis disease within the first two years of infection [40]. This underscores the importance of understanding the epidemiology of TB among Ethiopian children all over the country in order to identify TB hot spots and devise appropriate and better TB prevention, case identification, and control strategies. Another closely related and significantly relevant issue is the fact that children are usually infected with tuberculosis from close contact with actively coughing persons. The source case may be from household contacts, schools, or healthcare facilities [42]. The finding in the present study, in which slightly over half of the cases (51%) had known TB contacts, is also in line with this notion.

The nationwide incidence of bacteriologically confirmed childhood PTB in this study was 5.1 per 100,000 children. Interestingly, remarkable variations between regions to regions and regions ot the national childhood TB incidences were observed. The lowest bacteriologically confirmed childhood tuberculosis cases (2.45/100,000) incidence was observed from the Tigray region while the highest was from Gambella (11.57/100,000). Of course, differences in the incidence pattern among various regions within a country are not unique to our study; for example, region-to-region variation was reported from Taiwan (that ranged from 1.93 to 100,000 at the center region to 16.02 per 100,000 in the south region) [43]. Such region-to-region differences may be due to differences in the practice of TB prevention and control besides the actual differences in the burden of TB [44].

In this nationwide DR-TB survey, we found a 6.5% childhood TB drug resistance among the participant children. This rate is much lower than the one reported previously from China (28%) [45] but higher than the one reported from Zimbabwe (3%) [30]. Usually, drug-resistant tuberculosis in children originates from the transmission of DR-TB from adult patients in the community (predominantly household exposure) harboring high levels of DR [46]. This notion is supported by our finding that the children with DR-TB identified in this study had no previous history of TB treatment, demonstrating the presence of high adult-to-children DR-TB transmission in the community. On the other hand, the burden of childhood MDR TB cases was 1.3% in our result, which was lower than the rate reported from the drug resistance survey in South Africa (2.3%) [46] and China (4.6% ) [45]. The reason why childhood MDR-TB was higher in the two countries than the one observed in our study may be that the burden of MDR-TB in these countries is expected to be high as both of them are known to belong to high MDR-TB burden countries in contrast to Ethiopia, which has been currently removed by WHO, from the list of the 30 MDR-TB high burden countries [6].

In the present study, 80% (4 of the 5 cases) of DR-TB cases were observed among children in the age group 10–14 years, while none was detected among the under-five children, which is in agreement with the report indicated in a 2021 review [47]. This might reflect the possibility of under-detection among under-5 children probably due to difficulty to obtain appropriate sputum specimens from this population, which makes it one of the important ongoing challenges in the understanding of DR-TB in children.

Another interesting observation from our study is that childhood drug-resistant TB was higher among urban residents (3/5, 60%) compared to rural ones (2/5, 40%), in spite of the fact that TB incidence was higher among the rural than the urban resident children. But this was not the case in China, where high drug-resistant TB was observed more in rural (66.1%) than urban (33.9%) settings [45]. In Ethiopia, a previous study observed that some MDR-TB patients including adults diagnosed at rural health facilities could be referred to urban hospitals for better management, which may cause delays to patient diagnosis and treatment and consequently increase the risk of MDR-TB transmission [48]. Alternatively, it could be that TB treatment is more available in urban settings where there is a higher chance of developing drug resistance due to interment usage of drugs when patients fail to adhere to the appropriate treatment dose or duration (as is often the case or if the treatment outcome is unfavorable[49, 50]. Thus, children who have regular and prolonged contact with such adult patients could acquire drug-resistant TB from them; hence, more resistance rate among urban than rural children in this report [50].

### Strengths and Limitations of the study

Although the exclusion of presumably TB cases diagnosed based on clinical findings alone in this study may further muddle our understanding of childhood TB epidemiology in Ethiopia, our study, nevertheless, helps to peer through the level of bacteriologically confirmed childhood PTB cases and TB-drug sensitivity profile among this age group, which indirectly reflects DR-TB in the community. Both of these have not been previously addressed adequately. However, we were also challenged to find bacteriologically confirmed childhood PTB case notifications from previous studies to compare our study with since most childhood TB studies included both bacteriologically and clinically diagnosed TB cases.

An important limitation of this study, however, is that phenotypic DST was not performed for all of the 102 identified TB cases because of the difficulty encountered in resuscitating a significant number of the archived isolates (11 formerly culture-positive TB cases, and the remaining nine identified TB cases which were GeneXepertMTB/RIF positive but culture negative).

## Conclusion

Bacteriologically confirmed childhood PTB cases increased as the age of the children increased, which could probably be linked to a better detection rate because of the children’s ability to produce the right sputum samples. However, remarkable variations were observed between the different regions. Most of the bacteriologically confirmed childhood PTB and DR-TB detected were from newly treated children. Also, rural children were more affected by TB than their urban counterparts, while DR-TB was more among urban residents than rural children. This warrants a further well-designed survey that would enable us to identify TB transmission hot spots in the country.

### Electronic supplementary material

Below is the link to the electronic supplementary material.

Supplementary Material 1

## Data Availability

The data that support the findings of this study are available from the corresponding author Yeshiwork Abebaw.

## Abbreviations

CNRs: Case Notification Rates
DST: Drug susceptibility testing
DR-TB: Drug Resistant TB
EPHI: Ethiopian Public Health Institute
MDR-TB: Multi-Drug Resistance Tuberculosis
MGIT: Mycobacterium Growth Indicator Tube
LJ: Lowenstein-Jensen medium
OR: Odds ratios
PTB: Tuberculosis
SNNPR: Southern Nations Nationalities & Peoples Region
WHO: World Health Organization
