# Supplementary material for: Pulmonary tuberculosis case notification and burden of drug resistance among children under 15 years of age in Ethiopia: sub-analysis from third-round drug resistance tuberculosis survey
Source: BMC Pediatr. 2023 Aug 24;23:418. doi: 10.1186/s12887-023-04240-6 (PMC10463301; doi:10.1186/s12887-023-04240-6)
Supplement: Supplementary file 1 — Supplementary Material 1 [file 12887_2023_4240_MOESM1_ESM.docx]

| No | Health facility name | Region code | Region | |
| --- | --- | --- | --- | --- |
| 1 | Axum Health Center | 1 | Tigray | |
| 2 | Kasech Health Center | 1 | Tigray | |
| 3 | Nifasilik Lafito wereda 9 Health Center | 10 | Addis Ababa | |
| 4 | Zenbaba General Hospital | 10 | Addis Ababa | |
| 5 | Aymba Health Center | 3 | Amhara | |
| 6 | Azezo Health Center | 3 | Amhara | |
| 7 | Ambagiworgis Health Center | 3 | Amhara | |
| 8 | Seladinigay Health Center | 3 | Amhara | |
| 9 | Harbu Health Center | 3 | Amhara | |
| 10 | Lalibela Health Center | 3 | Amhara | |
| 11 | Eteya Health Center | 4 | Oromia | |
| 12 | Alem Gena Health Center | 4 | Oromia | |
| 13 | Semar Health Center | 4 | Oromia | |
| 14 | Anole Sodu Health Center | 4 | Oromia | |
| 15 | Anfele kola Health Center | 4 | Oromia | |
| 16 | Dogu Health Center | 4 | Oromia | |
| 17 | Garamulata Hospital | 4 | Oromia | |
| 18 | Girawa Health Center | 4 | Oromia | |
| 19 | Harawach Health Center | 4 | Oromia | |
| 20 | Jaja Health Center | 4 | Oromia | |
| 21 | Welenchiti Health Center | 4 | Oromia | |
| 22 | Bandira Health Center | 4 | Oromia | |
| 23 | Fincha Health Center | 4 | Oromia | |
| 24 | Fincha Sugar Factory Health Center | 4 | Oromia | |
| 25 | Hurumu Health Center | 4 | Oromia | |
| 26 | Harawa Gatira Health Center | 4 | Oromia | |
| 27 | Harawa Jimate Health Center | 4 | Oromia | |
| 28 | Ada Bonya Health Center | 4 | Oromia | |
| 29 | Ejere Health Center | 4 | Oromia | |
| 30 | Bantu Health Center | 4 | Oromia | |
| 31 | Roba Health Center | 4 | Oromia | |
| 32 | Robe Ashoka Health Center | 4 | Oromia | |
| 33 | Sire Shifa Health Center | 4 | Oromia | |
| 34 | Wabe Health Center | 4 | Oromia | |
| 35 | Ela Dima Health Center | 4 | Oromia | |
| 36 | Garba Health Center | 4 | Oromia | |
| 37 | Ijefera Health Center | 4 | Oromia | |
| 38 | Wachu Gile Health Center | 4 | Oromia | |
| 39 | Gorba Health Center | 4 | Oromia | |
| 40 | Chalia Health Center | 4 | Oromia | |
| 41 | Guliso Health Center | 4 | Oromia | |
| 42 | Kebri Beyah Health Center | 5 | Somali | |
| 43 | Bebeka Health Center | 7 | Southern Nations, Nationalities, and Peoples' Region |  |
| 44 | Kitte Health Center | 7 | Southern Nations, Nationalities, and Peoples' Region |  |
| 45 | Chencha Hospital | 7 | Southern Nations, Nationalities, and Peoples' Region |  |
| 46 | Hedere Health Center | 7 | Southern Nations, Nationalities, and Peoples' Region |  |
| 47 | Wonago Health Center | 7 | Southern Nations, Nationalities, and Peoples' Region |  |
| 48 | Halaba Health Center | 7 | Southern Nations, Nationalities, and Peoples' Region |  |
| 49 | Hawasa Referral Hospital | 7 | Southern Nations, Nationalities, and Peoples' Region |  |
| 50 | Kereda Health Center | 7 | Southern Nations, Nationalities, and Peoples' Region |  |
| 51 | Kelle District Hospital | 7 | Southern Nations, Nationalities, and Peoples' Region |  |
| 52 | Miridicha Health Center | 7 | Southern Nations, Nationalities, and Peoples' Region |  |
| 53 | Mejo Health Center | 7 | Southern Nations, Nationalities, and Peoples' Region |  |
| 54 | Olonso Keka Health Center | 7 | Southern Nations, Nationalities, and Peoples' Region |  |
| 55 | Motto Health Center | 7 | Southern Nations, Nationalities, and Peoples' Region |  |
| 56 | Dagia Health Center | 7 | Southern Nations, Nationalities, and Peoples' Region |  |
| 57 | Arada Gale Health Center | 7 | Southern Nations, Nationalities, and Peoples' Region |  |
| 58 | Wondo Genet Kella Health Center | 7 | Southern Nations, Nationalities, and Peoples' Region |  |
| 49 | Gebriber Health Center | 7 | Southern Nations, Nationalities, and Peoples' Region |  |
| 60 | Sodo Chiristian Hospital | 7 | Southern Nations, Nationalities, and Peoples' Region |  |
| 61 | Gambella Health Center | 8 | Gambella | |
| 62 | Jugol Hospital | 9 | Harari | |

Healthcare Facilities information
